# Novel nanocomposite-superlattices for low energy and high stability nanoscale phase-change memory

**DOI:** 10.1038/s41467-023-42792-4

**Published:** 2024-01-22

**Authors:** Xiangjin Wu, Asir Intisar Khan, Hengyuan Lee, Chen-Feng Hsu, Huairuo Zhang, Heshan Yu, Neel Roy, Albert V. Davydov, Ichiro Takeuchi, Xinyu Bao, H.-S. Philip Wong, Eric Pop

**Affiliations:** 1 Department of Electrical Engineering, Stanford University, Stanford, CA USA; 2 Corporate Research, Taiwan Semiconductor Manufacturing Company (TSMC), Hsinchu, Taiwan; 3 Materials Science and Engineering Division, National Institute of Standards and Technology, Gaithersburg, MD USA; 4 Theiss Research, Inc., La Jolla, CA USA; 5 Department of Materials Science and Engineering, University of Maryland, College Park, MD USA; 6 School of Microelectronics, Tianjin University, Tianjin, China; 7 Corporate Research, Taiwan Semiconductor Manufacturing Company (TSMC), San Jose, CA USA; 8 Department of Materials Science & Engineering, Stanford University, Stanford, CA USA; 9 Precourt Institute for Energy, Stanford University, Stanford, CA USA

**Keywords:** Electrical and electronic engineering, Electronic devices

## Abstract

Data-centric applications are pushing the limits of energy-efficiency in today’s computing systems, including those based on phase-change memory (PCM). This technology must achieve low-power and stable operation at nanoscale dimensions to succeed in high-density memory arrays. Here we use a novel combination of phase-change material superlattices and nanocomposites (based on Ge_4_Sb_6_Te_7_), to achieve record-low power density ≈ 5 MW/cm^2^ and ≈ 0.7 V switching voltage (compatible with modern logic processors) in PCM devices with the smallest dimensions to date (≈ 40 nm) for a superlattice technology on a CMOS-compatible substrate. These devices also *simultaneously* exhibit low resistance drift with 8 resistance states, good endurance (≈ 2 × 10^8^ cycles), and fast switching (≈ 40 ns). The efficient switching is enabled by strong heat confinement within the superlattice materials and the nanoscale device dimensions. The microstructural properties of the Ge_4_Sb_6_Te_7_ nanocomposite and its high crystallization temperature ensure the fast-switching speed and stability in our superlattice PCM devices. These results re-establish PCM technology as one of the frontrunners for energy-efficient data storage and computing.

## Abstract (web summary)

Data-centric applications benefit from dense, low-power memory. Here the authors use a combination of chalcogenide superlattices and nanocomposites to achieve low switching voltage (0.7 V) and fast speed (40 ns) in 40-nm-scale phase-change memory.

## Introduction

The rapid growth of big-data, high performance computing, and numerous data-centric artificial intelligence applications have inspired continued demand for robust and low-power nonvolatile memory^1–5^. Among emerging technologies, phase-change memory (PCM) based on chalcogenides could bridge the performance gap between existing data storage solutions such as flash (nonvolatile, but relatively slow) and dynamic random-access memory (fast, but volatile)^6–8^. In addition, PCM also benefits from large memory window ( > 100× ratio between resistance states) and multilevel operation, which are useful for brain-inspired computing applications^4,9–12^.

PCM based on traditional phase-change materials like Ge_2_Sb_2_Te_5_ (GST225) is known to suffer from high switching power and resistance drift, i.e., gradual change of its resistance states over time^13,14^. Recent progress on PCM devices has focused on lowering their reset energy^15–19^, however the on/off ratio^17,19^, endurance^17–19^, uniformity and process compatibility^15,16^ need improvement. Some efforts^20–22^ have also increased the PCM speed, but at the expense of reduced thermal stability^20^, larger set voltage^20,21^ or larger reset current^22^. In recent years, phase change materials arranged in superlattice (SL) stacks with alternating layers of GeTe/Sb_2_Te_3_^12,23–27^, TiTe_2_/Sb_2_Te_3_^10,28^, GeSb_2_Te_4_/Sb_2_Te_3_^29^, and Sb_2_Te_3_/GST225^30,31^ have enabled lower switching current and resistance drift of PCM, due to structural and electro-thermal confinement caused by van der Waals (vdW) interfaces within such superlattices^25,32–34^. However, to-date SL materials have not been optimized for the well-known trade-off between speed and stability (especially at higher temperatures) of PCM devices^9,20,35^, while SL memory cells have not yet been demonstrated with nanoscale dimensions. In other words, can SL-based PCMs maintain advantages as they approach the limits of (size) scaling, or is their performance curtailed by fundamental trade-offs?

To probe these limits, here we demonstrate ≈ 40 nm nanoscale PCM devices with the first superlattices based on Ge_4_Sb_6_Te_7_ (GST467), a new nanocomposite^36^ with higher crystallization and lower melting temperature than traditional PCM materials, consisting of epitaxial SbTe nanoclusters within a Ge-Sb-Te matrix^37^. These SbTe nanoclusters serve as a precursor for crystallization, also increasing the switching speed of GST467. Thus, by introducing GST467 into our superlattice PCM devices we *simultaneously* achieve record-low ≈ 5 MW/cm^2^ switching power density, ultra-low ≈ 0.7 V switching voltage, sub-1.5 pJ switching energy, fast switching speed (≈ 40 ns), low resistance drift with 8 resistance states and high endurance ( ≈ 2 × 10^8^ cycles). The efficient operation is enabled by strong heat confinement within the superlattice interfaces and nanoscale dimensions, while the unique microstructural properties of GST467 and its higher crystallization temperature facilitate the simultaneously faster switching speed and improved stability, going beyond the fundamental trade-off for PCM technology. From a materials perspective, this also represents the first time that the combination of bottom-up natural interfaces (in the nanocomposite) and top-down superlattice interfaces are simultaneously implemented in the same memory material, giving rise to the superior device performance.

## Results and discussion

**Fig. 1:**
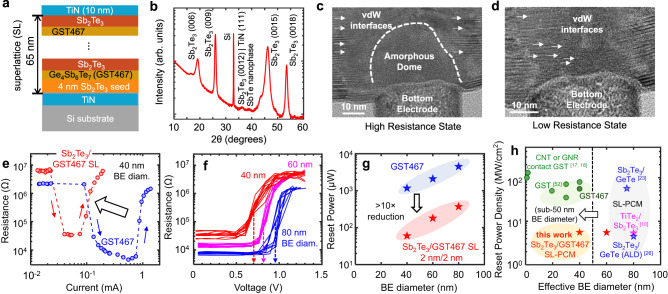
Superlattice phase-change memory (PCM) with GST467 nanocomposite. **a** Schematic, and **b** X-ray diffraction (XRD) of Sb_2_Te_3_/GST467 superlattice (SL) material stack on a TiN (20 nm thick)/Si substrate showing the polycrystallinity of the as-deposited SL. TEM cross-sections of **c** a nanoscale mushroom-cell device with 40 nm BE diameter in the high resistance state (HRS) and **d** a similar device in the low resistance state (LRS). Both devices and the superlattice films in **b** had 2/2 nm/nm Sb_2_Te_3_/GST467 superlattices, and both device TEMs were taken after ≈ 5000 electrical cycles. Dashed line in **c** outlines the amorphous region of the SL (in HRS) on top of the BE, surrounded by vdW-like SL interfaces (small arrows). VdW-like interfaces are restored throughout the device in the LRS in **d**, in agreement with previous reports on other SL-PCM^24,38^. **e** Measured dc read resistance vs. current, showing ≈ 10x reduction of reset current for superlattice PCM compared to control GST467 PCM (both with 40 nm BE diameter). Small arrows show the transitions from HRS to LRS and from LRS to HRS. **f** Read resistance vs. voltage for superlattice PCM devices with varying BE diameters (from 40 nm to 80 nm) showing sub-1 volt switching of our PCM devices. For each device, 10 different cycles are shown. Reset voltage (marked by colored dashed arrows) is defined as the voltage needed for a ≈ 10× resistance increase from LRS. **g** Reset power scales with BE diameter for both our superlattice PCM and control GST467 PCM, as expected (see resistance vs. reset power in Fig. S4b). Superlattice-like PCM devices show >10x reduction of reset power across different BE diameters, down to 40 nm. **h** Reset power density for various sub-100 nm PCM technologies^10,17,18,23,26,51,52^.This work enables the lowest reset power density to-date among nanoscale PCMs with sub-50 nm diameters. Here GST refers to Ge_2_Sb_2_Te_5_.

As shown in Fig. 1a, we deposited the superlattice material stacks either onto TiN films (for x-ray analysis) or onto TiN bottom electrodes (for mushroom-cell PCM devices). These superlattices consist of 15 periods of alternating layers of Sb_2_Te_3_ (≈ 2 nm) and GST467 (≈ 2 nm), sputtered at 180 °C followed by a 15-min in-situ anneal at 200 °C (see details in Methods: Materials deposition). We cap the films with TiN (10 nm) or TiN/Pt (10/10 nm/nm) top electrodes sputtered without breaking vacuum to complete the fabrication of our memory devices. Our mushroom-cell PCM devices have bottom electrode (BE) diameters between 40 nm and 80 nm.

X-ray diffraction (XRD) spectra in Fig. 1b confirm the polycrystallinity of our as-deposited Sb_2_Te_3_/GST467 superlattice film, with the same deposition conditions as our PCM devices. The sharp out-of-plane XRD peaks of Sb_2_Te_3_ correspond to the highly oriented SL layers parallel to the substrate. The same XRD figure also shows the presence of SbTe nanophase (from the GST467 nanocomposite material^36^). The transmission electron microscope (TEM) image in Fig. 1c shows the cross-section of one of our mushroom-cell devices (with ≈ 40 nm TiN BE) in the *high resistance* state (HRS) after ≈ 5000 electrical cycles, revealing an amorphous dome surrounded by preserved vdW-like interfaces (zoomed-in TEM and diffraction pattern in supplementary Fig. S1). Figure 1d displays the TEM cross-section of another mushroom-cell PCM device (also with ≈ 40 nm BE diameter) in the *low resistance* state (LRS) after ≈ 5000 electrical cycles, showing the presence of SL interfaces and vdW-like gaps (zoomed-in TEM in supplementary Fig. S2a). Thus, the vdW interfaces in our nanoscale superlattice PCM devices are sufficiently restored in the LRS after electrical cycling, which agrees with the previous literature for superlattice PCM with different materials and larger BE diameters^24,25,38^. The unoperated regions of the superlattice also show vdW-like interfaces (zoomed-out TEM in Supplementary Fig. S2b), including some atomic reconfiguration known to occur during deposition kinetics^39^.

Resistance (*R*) versus current (*I*) measurements in Fig. 1e show nearly an order of magnitude reduction of reset (switching) current in our well-cycled ( > 5000 times) Sb_2_Te_3_/GST467 superlattice device compared to control GST467 devices with same total film thickness ( ≈ 65 nm) and BE diameter ( ≈ 40 nm). For reset programming (LRS to HRS), we used 1/20/1 ns pulses and for set (HRS to LRS), we used pulses with 1/30/50 ns rise/width/fall time. Resistance states were read with a 50-mV direct current (dc) bias, and the measurement setup was further detailed elsewhere^12,40^. Sb_2_Te_3_/GST467 superlattice devices show higher LRS than control GST467 devices due to larger cross-plane electrical resistivity of the superlattice, caused by the internal vdW-like interfaces^33^. These interfaces also enable substantial heat confinement, leading to the significant reduction of reset current in the PCM with high-quality superlattices (here Sb_2_Te_3_/GST467), as detailed in earlier studies^25,32–34,38^. We also demonstrated similar behavior in a different superlattice (Sb_2_Te_3_/GST225) on a ≈ 40 nm bottom electrode in Supplementary Fig. S3. Although both GST467- and GST225-based superlattice PCMs with ≈ 40 nm BE diameter (smallest to date) show low reset current, the former has additional advantages of simultaneously fast switching and better thermal stability, as we will explore below.

Measured *R* vs. voltage (*V*) in Fig. 1f reveals sub-1 V switching for our Sb_2_Te_3_/GST467 superlattice PCM with varying BE diameters, from 40 nm to 80 nm. For the ≈ 40 nm devices, the reset voltage (*V*_reset_) is ≈ 0.7 V, the lowest to-date demonstrated in PCM technology. Sub-1 V operation makes this superlattice PCM compatible with modern logic processors^41^, which can enable embedded memory-logic integration for high-performance computing and Internet of Things^42,43^.

In Supplementary Fig. S4a we demonstrate that the reset current of the same set of devices scales properly (here by a factor of four) even at nanoscale dimensions, as we reduce the BE diameter from ≈ 80 nm to ≈ 40 nm. The lowest reset current is *I*_reset_ ≈ 85 µA in our ≈ 40 nm devices, and this can be further reduced by downscaling the BE diameter, as explored with electro-thermal simulations in Supplementary Fig. S5. The reset power, *P*_reset_, is obtained from *R* vs. power (*P*) (Supplementary Fig. S4b) and scales with BE diameter for both Sb_2_Te_3_/GST467 superlattice PCM and control GST467 devices as shown in Fig. 1g. The same Fig. 1g also displays >10× reduction of reset power for Sb_2_Te_3_/GST467 superlattice devices vs. control GST467 across all BE diameter devices in this work.

Our ≈ 40 nm Sb_2_Te_3_/GST467 superlattice PCM devices display *P*_reset_ of ≈ 60 µW, which can be further reduced by downscaling the BE diameter below 40 nm. Adjusted by the BE area, the corresponding switching power density is ≈ 5 MW/cm^2^, an order of magnitude lower than any comparable sub-50 nm diameter PCM devices reported to date (Fig. 1h). Supplementary Fig. S6 displays the scaling trends of reset power vs. BE diameter, showing how the reset power could be reduced below 10 μW in high-density superlattice PCM devices with critical dimension below ≈ 10 nm.

**Fig. 2:**
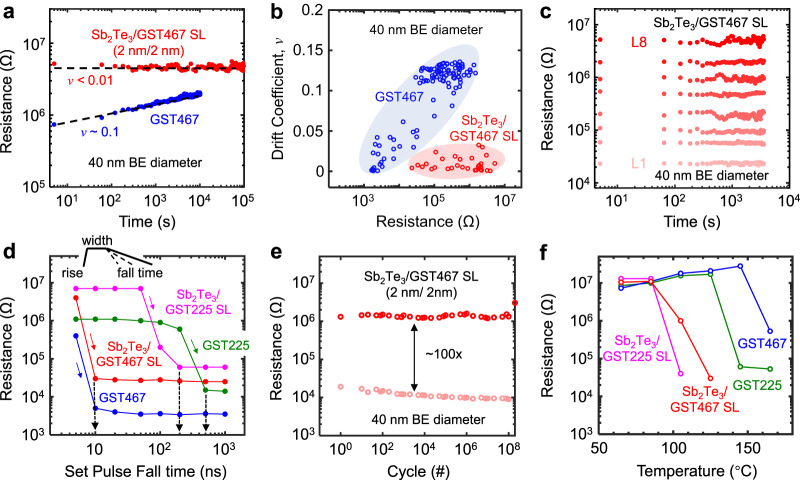
Resistance drift, speed, reliability, and endurance of GST467-based superlattice PCM. **a** High resistance state (HRS) vs. time, showing low drift in Sb_2_Te_3_/GST467 superlattice PCM vs. control GST467, both devices with ≈ 40 nm BE diameter. Dashed lines are fit to *R*(*t*) ∼ (*t*/*t*_0_)^*v*^, where *v* is the drift coefficient, *t* is the time after programming, and *t*_0_ is a constant. **b** Drift coefficient *v* as a function of resistance state for the same superlattice (SL, red symbols) and non-superlattice (blue symbols) devices. **c** Eight resistance states with low drift maintained > 1 hour in our GST467-based superlattice PCM with 40 nm diameter, enabling a multi-level cell with up to 3 bits. **d** Effect of fall times on set transition for four types of PCM, as labeled. All pulses have 1 ns rise time and 30 ns widths, and all devices have 40 nm diameter. The minimum fall times to reach the LRS are marked with black dashed arrows. The GST467-based devices can switch with >10x shorter set fall time ( ≈ 10x faster switching) compared to control devices based on GST225, for both superlattice and non-superlattice PCM. Set voltages for Sb_2_Te_3_/GST467, Sb_2_Te_3_/GST225, GST467 and GST225 are 0.65 V, 0.8 V, 1.2 V, and 1.3 V, respectively. **e** Endurance up to 2 × 10^8^ cycles measured for our GST467-based superlattice PCM with 40 nm BE diameter, maintaining a 100× resistance window. **f** High-temperature HRS stability of our superlattice PCM compared to control devices. After programming to HRS, devices were annealed for 30 min at successively higher temperatures. We reached each of the upper resistance levels by single-shot reset pulses from the LRS. DC resistances are measured back at room temperature after each annealing event. The higher crystallization temperature of GST467 enables higher temperature stability of PCM based on it. The larger HRS ≈ 10 MΩ in Fig. 2f (vs. Figure 1e and Fig. 2e) is due to differences in the amorphous volume originating from the different pulsing schemes. In addition, fabrication-induced variations between devices can also contribute to observed differences in HRS. All resistances in (**a-f**) are measured with 50 mV dc bias. Devices in **a**–**d** and **f** were well-cycled ( > 5000 cycles) before measurements.

We now turn to features of resistance drift, speed, and stability in our superlattice PCM. Resistance drift is already known to be low in other types of superlattice PCM (with larger diameter), based on reports from our group^31^ and others^10,27^. Here we confirm that low resistance drift (with coefficient *v* < 0.01) is maintained in our nanoscale ≈ 40 nm Sb_2_Te_3_/GST467 superlattice devices (Fig. 2a) compared to control PCM based on GST467 (*v* ≈ 0.1). We further find that the low resistance drift of our superlattice PCM is maintained across different resistance states (Fig. 2b). Thus, we are able to demonstrate eight distinct resistance states with low drift in our ≈ 40 nm superlattice PCM devices (Fig. 2c and Supplementary Fig. S7), which is promising for high-density multi-level data storage.

In terms of switching speed, PCM devices are usually limited by the set transition, from HRS to LRS. Here, we find that our GST467-based superlattice PCMs are > 3× faster than other superlattice PCM types and > 10× faster than common (i.e., single-material) PCM devices (see details below). To understand where the benefits come from, in Fig. 2d we compare our GST467-based superlattice PCM with GST225-based superlattice PCM and with common memory cells using either GST225 or GST467. In this figure, the rise time and pulse widths are fixed at 1 ns and 30 ns, respectively, while varying the pulse fall time. Our GST467-based superlattice PCM devices are faster ( ≈ 40 ns) than GST225-based superlattice devices ( ≈ 200 ns) for same 40 nm BE diameter. Our previous reports^24,30^ on both GST225-based^30^ and GeTe-based^24^ superlattice PCM showed similar set switching speed at a similar voltage. Thus GST467-based superlattice PCM presents an advantage of faster switching speed over other superlattice-type PCM devices.

We observe that the faster switching speed of GST467-based superlattice PCM originates from the intrinsically faster speed of GST467 ( ≈ 40 ns) compared to GST225 PCM ( ≈ 500 ns) and GeTe ( ≈ 220 ns)^24^ based superlattice devices. We note that an even faster set speed could be achieved, however at the expense of a larger set voltage. Previous reports^36,37^ on GST467 nanocomposite confirmed the presence of the SbTe nanophase (also evident from our TEMs in Supplementary Fig. S8 and Supplementary Fig. S9a) grown coherently with the cubic Ge-Sb-Te matrix along {111}_cubic_ crystallographic planes. The thickness of two-atom-thick SbTe^36,37^ in the (001) direction is ≈ 0.35 nm (Supplementary Fig. S8e); thus the SbTe nanoclusters are still expected to be present within the ≈ 2 nm Ge_4_Sb_6_Te_7_ thin layers across the superlattice stack. The presence of SbTe nanophase within the GST467-based superlattice stack is also confirmed in our XRD measurements (Fig. 1b). Such SbTe nanoclusters act as nucleation sites and enable faster switching in GST467-based superlattice PCM. Moreover, a similarity in the bonding between amorphous and crystalline GST467^37^ also indicates that a structure in the amorphous state serves as a precursor for the faster crystallization^21^ of this material. We further note that the microstructure of GST467 in our superlattice Sb_2_Te_3_/GST467 devices can also be influenced by the adjacent Sb_2_Te_3_ layers, which could introduce some structural frustration and help control the PCM device performance.

Cycling measurements in Fig. 2e reveal that our ≈ 40 nm superlattice PCM devices can simultaneously achieve a large resistance window ( > 100×) and large endurance over >10^8^ switching cycles. The robustness of the simultaneously low reset voltage and large on/off ratio in our superlattice devices is further displayed in Supplementary Fig. S10. Resistance vs. temperature measurements in Fig. 2f demonstrate higher temperature stability of the HRS of GST467-based superlattice PCM, compared to our control superlattice devices with GST225, thanks to the better thermal stability of GST467 vs. GST225. This is attributed to a higher crystallization temperature in GST467 ( ≈ 200 °C) vs. GST225 ( ≈ 150 °C), confirmed by temperature dependent XRD (Supplementary Fig. S9a) and sheet resistance (Supplementary Fig. S9b) of both materials.

**Fig. 3:**
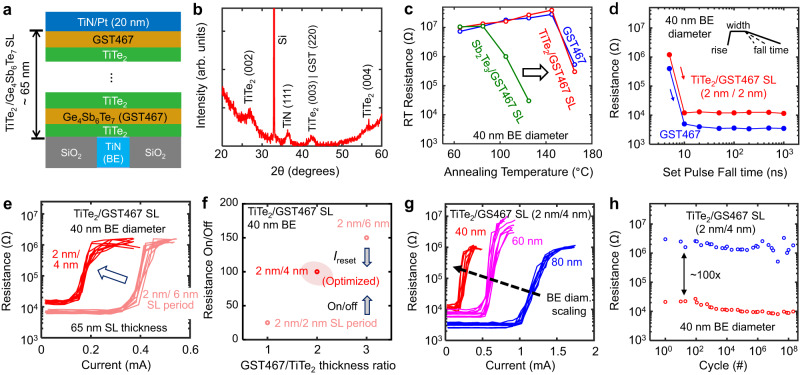
Superlattice devices with GST467 nanocomposite and TiTe_2_ thermal barriers. **a** Schematic of TiTe_2_/GST467 superlattice device, TiTe_2_ forming thermal barriers^28^ and GST467 as the phase-change layers. **b** XRD of TiTe_2_/GST467 superlattice (SL) on a TiN (20 nm thick)/Si substrate showing the polycrystallinity of the as-deposited SL. **c** Comparing the high-temperature stability of HRS in a TiTe_2_/GST467 superlattice device with a Sb_2_Te_3_/GST467 superlattice device and a GST467 control device (all with 40 nm BE diameter). The HRS of TiTe_2_/GST467 and control GST467 devices are similar, with no re-crystallization for >3 hours at 145 °C. Measurement protocols are described in Fig. 2f. Room temperature (RT) is 20 °C. **d** Effect of fall times on set transition for TiTe_2_/GST467 and control GST467 devices. Devices have 40 nm BE diameter, and all pulses have 1 ns rise time and 30 ns widths. Measurement protocols are described in Fig. 2d. Both device types show fast switching speed of ≈ 40 ns. **e** Effect of SL period thickness on device reset current, keeping SL thickness fixed ( ≈ 65 nm). We expect the GST467 layer to dominate the overall on/off ratio in the superlattice stack because TiTe_2_ itself has a small resistance on/off ratio^47^ (≈4). On the other hand, thinner GST467 layers lead to a reduction of reset current, as more internal interfaces enable better heat confinement^30^. **f** Effect of SL period thickness on device resistance on/off ratio. Low reset current and ≈ 100× resistance window are simultaneously achieved with the 2/4 nm/nm TiTe_2_/GST467 superlattice devices. **g** Scaling of reset current with BE diameter (from ≈ 40 to 80 nm) is maintained for TiTe_2_/GST467 superlattice devices. For reset programming (LRS to HRS), we used 1/20/1 ns pulses. **h** Endurance of our optimized 2/4 nm/nm TiTe_2_/GST467 superlattice PCM with 40 nm BE diameter (up to 2 × 10^8^ cycles), while maintaining a resistance on/off ratio of ≈ 100.

Supplementary Fig. S11 shows that the retention of our Sb_2_Te_3_/GST467 superlattice PCM is ≈ 10^5^ hours at 83 °C (close to the product-level requirement of 10^5 ^ hours at 85 °C^44^). The high-temperature retention of Sb_2_Te_3_/GST467 superlattice devices can still be limited by the lower crystallization temperature of Sb_2_Te_3_^45^. To enable even better temperature stability in our superlattice PCM, we next replaced the Sb_2_Te_3_ layers with a thermal barrier material of higher melting temperature, TiTe_2_^28,46^ and fabricated TiTe_2_/GST467 superlattice devices, as shown in Fig. 3a (schematic) and supplementary Fig. S12 (high resolution TEM). These films are deposited by sputtering, very similar to our Sb_2_Te_3_ layers, except for an in-situ annealing step at 300 °C (see further details in Methods: Materials deposition). XRD spectra in Fig. 3b confirm the out-of-plane features of the as-deposited TiTe_2_/GST467 superlattice film on a TiN/Si substrate, where the TiN surface is chosen to mimic the PCM bottom electrode composition. High-resolution TEM cross-sections of well-cycled ( ≈ 10^4^ times) TiTe_2_/GST467 superlattice PCM devices in the HRS and LRS are shown in Supplementary Fig. S13 and Fig. S14, respectively.

Temperature-dependent measurements of the HRS confirm the significantly higher temperature stability of TiTe_2_/GST467 superlattice devices compared to those based on Sb_2_Te_3_/GST467 (Fig. 3c). Supplementary Fig. S11 shows that the retention of our TiTe_2_/GST467 superlattice PCM is ≈ 10^5 ^h at 120 °C, promising for applications that require higher temperature retention^42^. Our TiTe_2_/GST467 superlattice PCM also maintains fast switching speed ( ≈ 40 ns) in devices with ≈ 40 nm bottom electrode (Fig. 3d). Therefore, our nanoscale TiTe_2_/GST467 devices offer simultaneously fast switching speed *and* higher temperature stability, by combining the unique properties of the GST467 nanocomposite with the thermal barrier properties of TiTe_2_, within a superlattice structure.

Electrical measurements of 40 nm BE diameter TiTe_2_/Ge_4_Sb_6_Te_7_ superlattice devices further show that both the reset current (Fig. 3e) and the resistance on/off ratio (Fig. 3f) can be simultaneously optimized by varying the thickness of the GST467 layer within an SL period (the TiTe_2_ layer is fixed at ≈ 2 nm and the total SL thickness is ≈ 65 nm). Thus, low switching current of ≈ 180 µA and resistance on/off ratio of ≈ 100 are simultaneously achieved in a 2/4 nm/nm TiTe_2_/GST467 superlattice device with 40 nm BE diameter, whereas *R* vs. *V* for the same device (Supplementary Fig. S15) confirms the sub-1 V switching operation with *V*_reset_ ≈ 0.85 V. We also note that the reset current measured here is ≈ 2× higher (for the same diameter) than for the Sb_2_Te_3_/GST467 devices (Fig. 1e and Supplementary Fig. S4a) due to the smaller LRS in TiTe_2_/GST467, which is attributed to the higher electrical conductivity of TiTe_2_^28,47^. Figure 3g displays the scaling of reset current with BE diameter (from ≈ 80 nm down to ≈ 40 nm) for 2/4 nm/nm TiTe_2_/GST467 superlattice devices and shows the clear pathway towards further lowering the reset current. Our optimized 2/4 nm/nm TiTe_2_/GST467 superlattice devices with ≈ 40 nm BE diameter also show good endurance for >10^8^ switching cycles, maintaining a resistance on/off ratio ≈ 100 (Fig. 3h).

The sharp vdW-like interfaces within the superlattice are responsible for the significant reduction of reset power in our SL-PCM. Previous studies^23,48^ had suggested that crystalline-to-crystalline transition through Ge atom movement may be responsible for switching in Sb_2_Te_3_/GeTe superlattice PCM. In contrast, our nanoscale superlattice PCM devices show a thermally-driven crystalline-to-amorphous transition (Fig. 1c, supplementary Fig. S1, Fig. S13, Fig. S16a, b). The low switching power originates from heat confinement of the vdW-like interfaces within the superlattice. We note that some interfacial reconfiguration between the superlattice layers can occur after electrical cycling, or during the delicate TEM sample preparation and imaging. However, van der Waals-like gaps appear sufficiently restored after cycling back to the LRS, enabling the heat confinement and low reset current in superlattice PCM^24,25^. Very recently, using nano-calorimetry^49^ we also found that the melting temperature of Sb_2_Te_3_/GST225 superlattices is ≈ 380 °C (240 °C lower than that of bulk GST225), providing additional insights into the low-power switching of these devices. Furthermore, a smaller active volume of the amorphous region (supplementary Fig. S1) compared to GST225 PCM^28^ can also contribute to the reduced switching power of our SL-PCM devices. The low energy switching of superlattice PCM in this work is further aided by the nanoscale device dimensions ( ≈ 40 nm BE diameter) compared to other superlattice PCM demonstrations^12,24,30^. Additional improvement in the switching energy of our superlattice PCM devices could be possible by further narrowing the reset pulse width^50^.

**Fig. 4:**
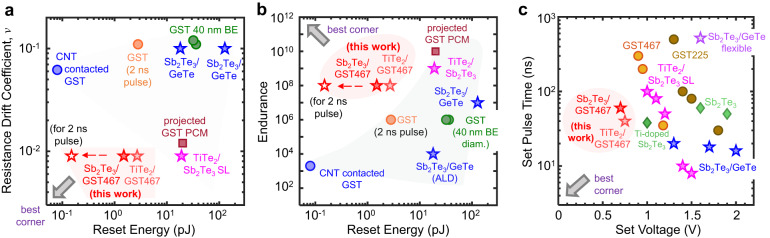
Benchmarking PCM technologies. **a** Resistance drift coefficient vs. reset energy, and **b** endurance vs. reset energy. Block arrows point to the desirable “best corners” with low resistance drift, high endurance, and low reset energy. Our GST467 nanocomposite-superlattice devices display some of the best overall characteristics, compared to all other existing PCMs^10,18,21,23,26,50–53,54^. The reset energy in our work (red filled star) is limited by our ~20 ns pulse width and instrumentation,^12,40^ which are not fundamental limits^13,50^. With 2 ns pulse widths^50^ the reset energy of our 40 nm superlattice PCM is projected (hollow red star) to reach ≈ 0.15 pJ. GST-based PCM with carbon nanotube (CNT) electrodes ( ≈ 1.7 nm diameter^18^) shows comparable reset energy to our superlattice PCM ( ≈ 40 nm diameter), but devices with CNT electrodes have limited endurance and high resistance drift (blue circles). This also shows that our reset energy can be reduced further, by decreasing the BE diameter. **c** Set pulse time vs. set voltage. The block arrow points to the desirable “best corner” with low set voltage and short set pulse time. Our GST467 nanocomposite-superlattice PCM is located near the best corner, compared to other existing PCMs^10,12,21,23,54,55^. To simplify notation, GST refers to the GST225 stoichiometry in the entire figure.

Finally, we compare our GST467-based superlattice PCM with previous demonstrations, including superlattice devices (of larger BE diameters)^10,18,23,26,50–53^, by plotting both drift coefficient (Fig. 4a) and endurance (Fig. 4b) vs. reset energy as well as switching speed vs. switching voltage (Fig. 4c). Our ultra-scaled 40 nm BE diameter devices demonstrate simultaneously low switching energy with large resistance on/off ratio, low resistance drift with multilevel operation, fast switching speed and high endurance, thus approaching the “best corners” of the benchmarking plots. We find a reset energy <1.5 pJ ( ≈ 60 µW reset power multiplied by 20 ns reset pulse, limited by our measurement instrument) in our ≈ 40 nm superlattice devices. Because PCM could be reset^50^ with pulse widths down to ≈ 2 ns, we estimate the reset energy for our smallest ( ≈ 40 nm) device could be as low as <0.15 pJ (hollow red stars in Fig. 4a, b), which can be further reduced by scaling down the PCM device dimensions, beyond the records achieved in this work. Figure 4c shows the set time vs. set voltage trade-off (i.e., a smaller set time can be achieved at the expense of a larger set voltage) in PCM technology^9^. Our GST467 nanocomposite-superlattice devices are near the best corner, with low set voltage and short set pulse time compared to other PCM demonstrations using GST225, doped Sb_2_Te_3_^21,54^, and other superlattices^10,12,23,55^. Thus, the GST467-based superlattice PCM in this work offers a unique simultaneous advantage of faster switching speed and better retention over other superlattice-type (GeTe/Sb_2_Te_3_^12,23–27^, TiTe_2_/Sb_2_Te_3_^10,28^, GeSb_2_Te_4_/Sb_2_Te_3_^29^, and Sb_2_Te_3_/GST225^30,31^) PCM devices. Additionally, our nanoscale superlattice devices with the smallest dimensions to date ( ≈ 40 nm) for a superlattice technology on a CMOS-compatible substrate further ascertain the promise of this technology for future high-density and energy-efficient PCM.

Thus, our nanocomposite-based superlattice PCMs (both Sb_2_Te_3_/GST467 and TiTe_2_/GST467) exhibit significantly reduced reset energy, sub-1 V switching, lower resistance drift, and better endurance compared to those of traditional PCMs. The low reset energy, sub-1 V operation, and fast switching position them among the leading next-generation memory candidates for on-chip logic and memory heterogeneous integration^43,56,57^. In addition, we find TiTe_2_/GST467 has better retention at high temperatures and could be promising as embedded memory for automotive applications^42^. Meanwhile, Sb_2_Te_3_/GST467 with simultaneously large on/off ratio and low resistance drift is well-positioned for emerging analog computing applications^4,58^.

In summary, we demonstrated nanoscale superlattice (SL) phase-change memory devices down to ≈ 40 nm dimensions, based on Ge_4_Sb_6_Te_7_ nanocomposite, and achieved low switching energy ( ≈ 1.5 pJ), fast switching speed ( ≈ 40 ns), and good endurance ( > 10^8^ cycles). The low-power operation is enabled by strong heat confinement within the material superlattice, integrated with the nanoscale ≈ 40 nm bottom electrode. The robustness of our nanoscale devices is confirmed using three different superlattices: Sb_2_Te_3_/GST467, TiTe_2_/GST467, and Sb_2_Te_3_/GST225. Among these, the microstructural properties of GST467 enable faster switching, while its higher crystallization temperature leads to better thermal stability. This work provides key materials and engineering insights towards the design and optimization of energy-efficient PCM, and could inspire the industry-scale adoption of nanoscale superlattice phase-change materials for low-power and high-density storage.

## Methods

### Material deposition

Before the deposition of the superlattice (SL) materials, the bottom TiN surface was in-situ cleaned by Ar ion etching for 10 minutes using 50 W radio-frequency (RF) bias to remove any native oxide. For the deposition of the Sb_2_Te_3_/Ge_4_Sb_6_Te_7_ (GST467) SL, first, a ≈ 4 nm thick Sb_2_Te_3_ seed layer was deposited on the bottom TiN at room temperature (sputter chamber base pressure < 10^−7 ^Torr). Then, the temperature in the sputter chamber was raised to ≈ 180 °C at a rate of 10 °C/min. and 15 periods of GST467 ( ≈ 2 nm) and Sb_2_Te_3_ (≈ 2 nm) alternating layers were deposited at ≈ 180 °C followed by an annealing of the stack at 200 °C for 15 min to ensure better crystallinity (total SL stack thickness ≈ 65 nm). For the deposition of the GST467 layer we used 20 sccm Ar flow, 12 W dc power, 2 mTorr pressure while for sputtering Sb_2_Te_3_ we used 30 sccm Ar flow, 35 W rf power, 4 mTorr pressure. The period thickness was chosen based on our measurements of SL cross-plane thermal conductivity of a similar SL stack (Sb_2_Te_3_/GST225) to ensure low thermal conductivity (higher heat confinement) as well as low resistance drift^30,31^.

For the deposition of the TiTe_2_/GST467 superlattice, TiTe_2_ and GST467 alternating layers ( ≈ 65 nm SL thickness in total) were deposited on the bottom TiN at ≈ 180 °C followed by in-situ annealing at 300 °C for 30 min in the sputter chamber. TiTe_2_ layers were sputtered with 30 sccm Ar flow, 30 W rf power, 4 mTorr pressure, and for the deposition of the GST467 layer we used 20 sccm Ar flow, 12 W dc power, 2 mTorr pressure. For the optimization of the TiTe_2_/GST467 SL-PCM devices, we fabricated SLs with varying periods e.g., with 2/2 nm/nm, 2/4 nm/nm and 2/6 nm/nm of TiTe_2_/GST467.

### Device fabrication

After the deposition of the SL layers, we let the sputtering chamber cool down to room temperature and then deposit a ≈ 10 nm TiN capping layer in situ (reactive sputtering of Ti with N_2_; 30 sccm Ar, 15 sccm N_2_, 3 mTorr pressure at 100 W dc power for Ti). The TiN layer acts as a capping layer to protect the SL from oxidation and as part of the top electrode for the PCM devices. For the SL-PCM devices, we also subsequently deposit ≈ 10 nm Pt (25 sccm Ar, 2 mTorr pressure at 100 W dc power) at room temperature as part of the rest of the top electrode to complete the fabrication process.

### Supplementary information

Supplementary Information

Peer Review File

## Data Availability

All data needed to evaluate the conclusions in this paper are available within the paper and the Supplementary Information file.
