# Supplementary material for: Novel nanocomposite-superlattices for low energy and high stability nanoscale phase-change memory
Source: Nat Commun. 2024 Jan 22;15:13. doi: 10.1038/s41467-023-42792-4 (PMC10803317; doi:10.1038/s41467-023-42792-4)
Supplement: Supplementary file 1 — Supplementary Information [file 41467_2023_42792_MOESM1_ESM.pdf]

Supplementary Information

Novel Nanocomposite-Superlattices for Low Energy and High Stability Nanoscale Phase-Change Memory

Xiangjin Wu[1,†], Asir Intisar Khan[1,†], Hengyuan Lee[2], Chen-Feng Hsu[2], Huairuo Zhang[3,4], Heshan Yu[5,6], Neel Roy[1], Albert V. Davydov[3], Ichiro Takeuchi[5], Xinyu Bao[7], H.-S. Philip Wong[1], and Eric Pop[1,8,9,*]

[1]*Department of Electrical Engineering, Stanford University, Stanford, CA 94305, USA*

[2]*Corporate Research, Taiwan Semiconductor Manufacturing Company (TSMC), Hsinchu, Taiwan*

[3]*Materials Science and Engineering Division, National Institute of Standards and Technology, Gaithersburg, MD, 20899 USA*

[4]*Theiss Research, Inc., La Jolla, CA, 92037 USA*

[5]*Department of Materials Science and Engineering, University of Maryland, College Park, MD, 20742, USA*

[6]*School of Microelectronics, Tianjin University, Tianjin 300072, China*

[7]*Corporate Research, Taiwan Semiconductor Manufacturing Company (TSMC), San Jose, CA 95134, USA*

[8]*Department of Materials Science & Engineering, Stanford University, Stanford, CA 94305 USA*

[9]*Precourt Institute for Energy, Stanford University, Stanford, CA 94305, USA*

[†]These authors contributed equally to the work. *Email: epop@stanford.edu

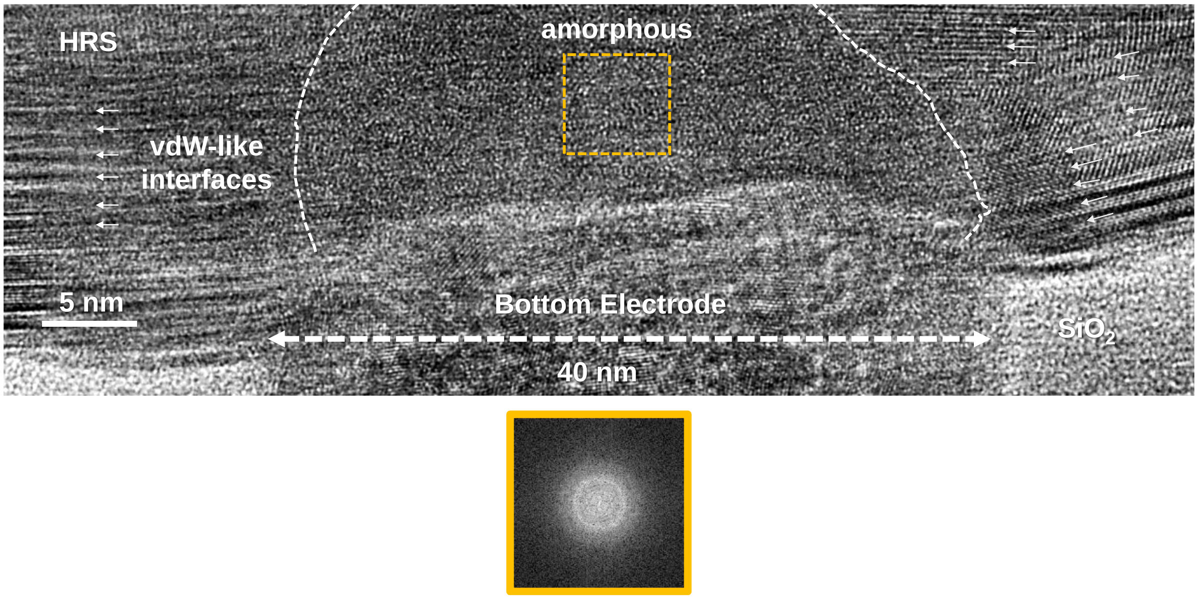

Supplementary Fig. S1 | High-resolution TEM cross-section of Sb_2Te_3/GST467 superlattice PCM device (zoomed-in version of Fig. 1c near BE) in the **high-resistance state** (HRS). The diffraction pattern corresponds to the region above the BE within the amorphous region. We infer the possible presence of nanocrystallites[1,2] within this amorphous region of Sb_2Te_3/GST467 superlattice, as evidenced by the diffraction rings (inset) which might further facilitate the fast-switching[2,3] of these devices.

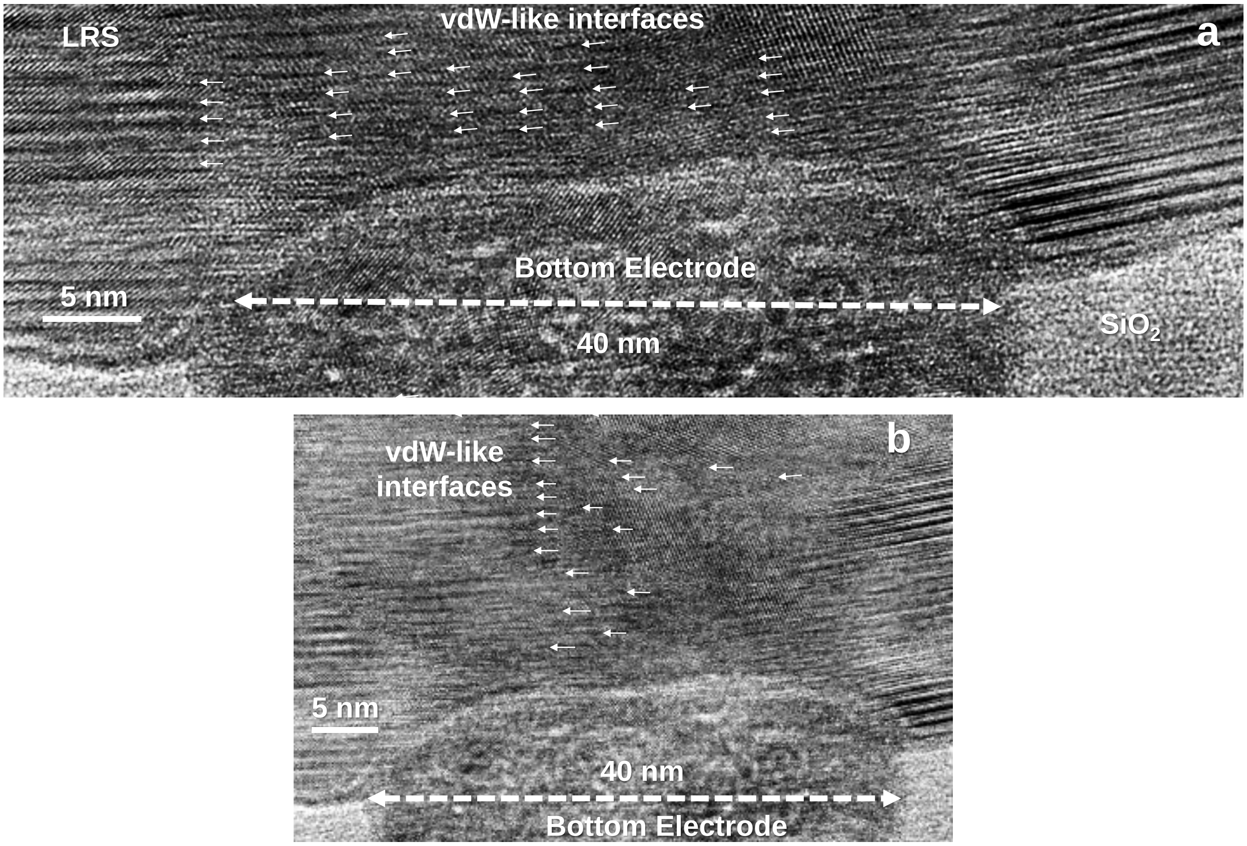

Supplementary Fig. S2 | a, High-resolution TEM cross-section of a 40 nm BE diameter Sb_2Te_3/GST467 superlattice PCM device (zoomed-in version of Fig. 1d near BE) in the **low-resistance state** (LRS). **b**, Zoomed-out TEM showing the unoperated region of the superlattice stack above the active superlattice area and the BE.

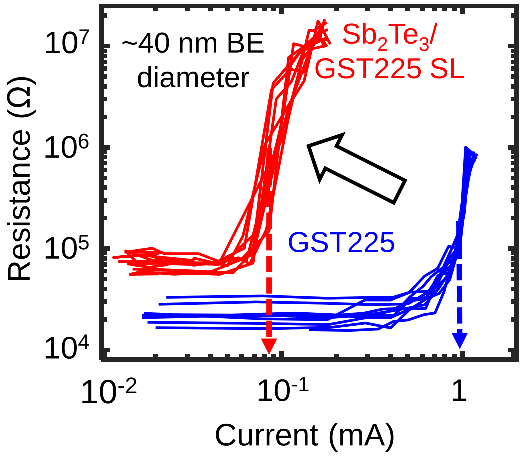

Supplementary Fig. S3 | Measured dc read resistance vs. current for Sb_2Te_3/GST225 superlattice (SL) PCM and control GST225 PCM device (both with 40 nm bottom electrode diameter). The SL-PCM device shows ≈ 10x reduction in the reset current compared to GST225 PCM (10 different cycles shown for each device). Reset current (marked by colored dashed arrows) is defined as the current needed for a ≈ 10× resistance change from LRS.

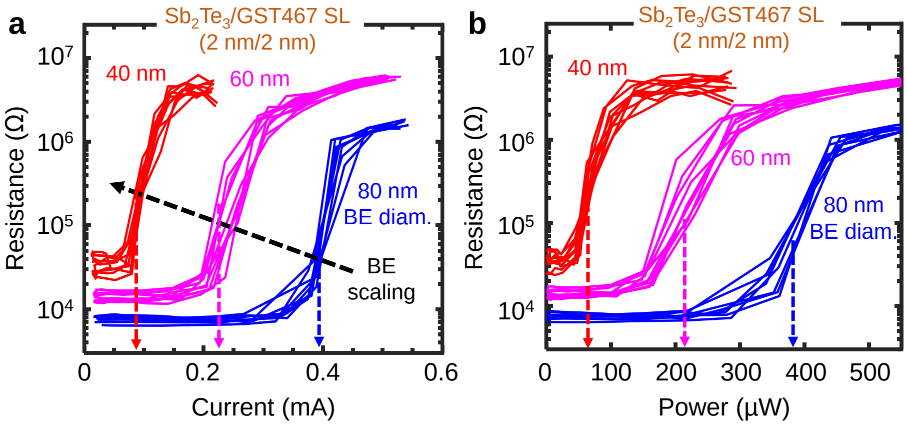

Supplementary Fig. S4 | Measured dc read resistance (*R*) vs. **a,** current (*I*), and **b,** power (*P*) for Sb_2Te_3/GST467 SL-PCM devices with varying BE diameter (from 80 nm down to 40 nm). 10 different cycles are shown for each device in both figures. Dashed colored arrows indicate the reset current (in **a**) and reset power (in **b**) for different BE diameter SL-PCM devices (also see **Fig. 1g**). *R* vs. *P* in **b** is calculated from *R* vs. *V* (**Fig. 1f**) and *R* vs. *I* (**a**).

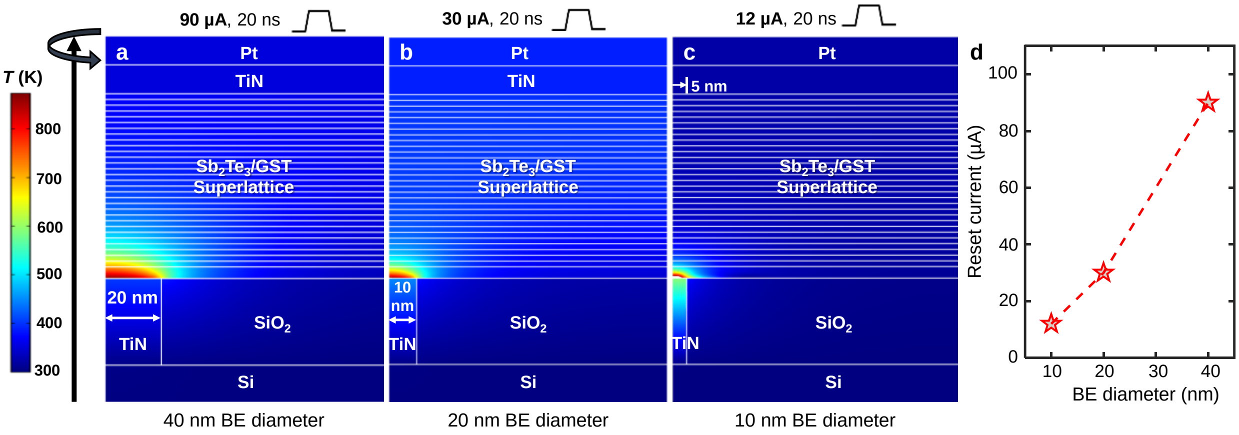

Supplementary Fig. S5 | Electro-thermal simulations. a-c, Temperature distributions after reset current pulses (20 ns) of varying magnitudes in Sb_2Te_3/GST mushroom cell SL-PCM for different bottom electrode (BE) diameters: **a**, 40 nm **b**, 20 nm, and **c**, 10 nm. The simulated device structure in **a** is same as the fabricated SL-PCM shown in Fig. 1c,d. The magnitude of the reset current pulse is defined as the minimum current needed for the simulated device temperature to reach ≈ 890 K (melting temperature of the constituent materials within the SL). The left edge is the axis of cylindrical symmetry, and the vertical and horizontal scales are unequal. **d,** Simulated reset current in Sb_2Te_3/GST SL-PCM as a function of BE diameter, showing that reset current is expected to decrease with decreasing BE diameter, as expected for thermally driven PCM technology.

For the simulations, we used the electrical resistivity and the thermal conductivities of Sb_2Te_3/GST(x:y:z) superlattices (SLs)[4]. We note that the SL electro-thermal properties responsible for heat confinement are primarily dependent on the numerous vdW interfaces within the SL stack, not on the thermal conductivity or electrical resistivity of the individual constituent materials[4-6]. Thus, similar temperature distribution profiles are expected for Sb_2Te_3/GST467 superlattices and will not alter the main outcome of the simulation (i.e., decreasing reset current with decreasing BE diameter). The simulation parameters and methods are further detailed in Refs. [5,7].

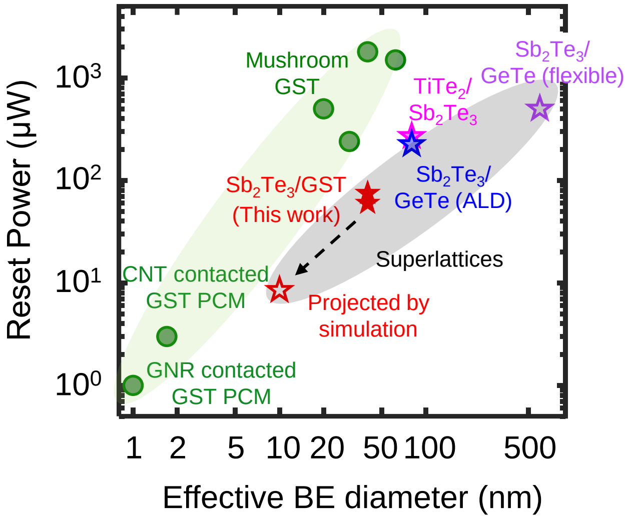

Supplementary Fig. S6 | Reset power vs. effective BE diameter for various PCM technologies. Superlattice-like PCM devices demonstrate ≈ 10x smaller reset power across different BE diameters. Our Sb_2Te_3/GST467 and Sb_2Te_3/GST225 superlattice PCM devices (red stars) show the smallest reset power to-date in a mushroom device geometry. Following the projection by simulation, we estimate that sub-8 nm BE diameter will lead to superlattice PCM with lower reset power than PCM based on simple GST225 with carbon nanotube (CNT) electrodes, which have ≈ 1.7 nm diameter.

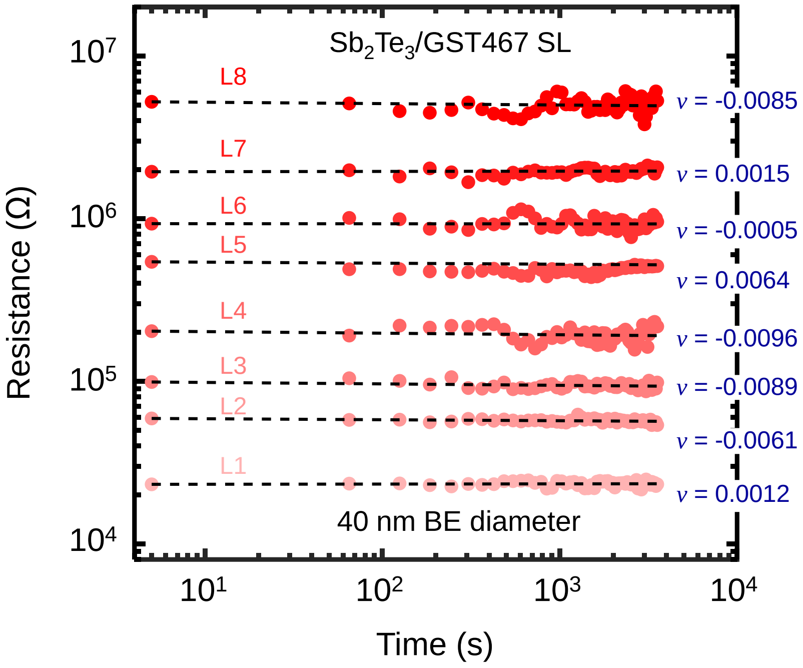

Supplementary Fig. S7 | Extraction of resistance drift coefficients of eight different resistance states of Sb_2Te_3/GST467 superlattice PCM. Dashed lines are fit to $R(t) \sim (t/t_0)^{\nu}$, where ν is the drift coefficient, t is the time after programming, and t_0 is a constant. All eight states have resistance drift coefficient $\nu < 0.01$.

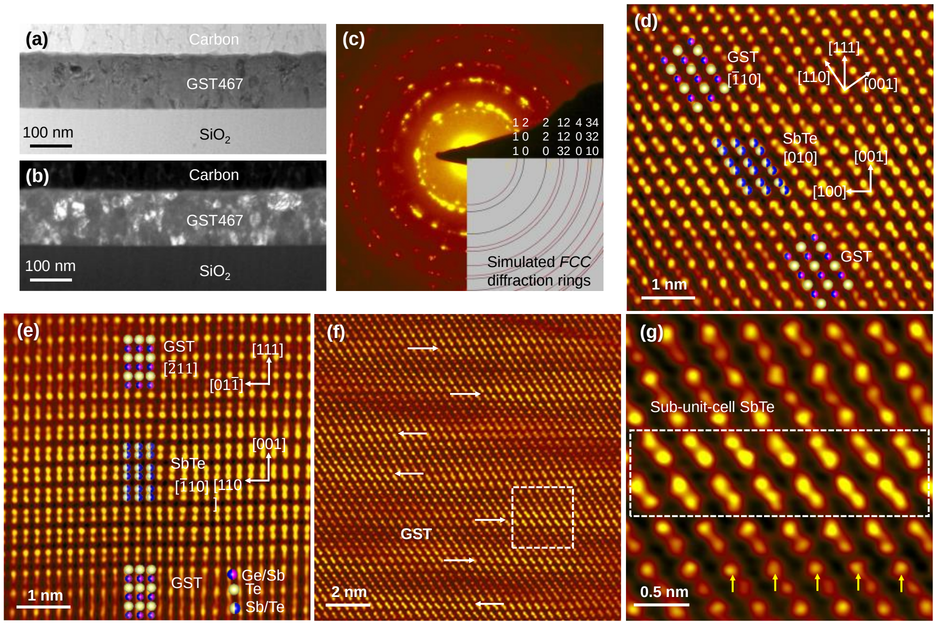

Supplementary Fig. S8 | a, A typical bright-field TEM image and **b,** the corresponding dark-field TEM image showing polycrystalline grains in a GST467 thin film. The TEM imaging is performed on a ~130 nm thick GST467 sample on a SiO_2/Si substrate. **c,** Selected area electron diffraction pattern from the GST467 film showing that the polycrystalline rings can mostly be indexed with FCC structure. **d,** Atomic resolution high-angle annular dark-field imaging (HAADF)-STEM images superimposed with projected atomic models showing the rhombic SbTe nanophase coherently precipitates in the cubic GST matrix with well-defined crystallographic orientation along $[\bar{1}10]_C$ zone-axis demonstrating $[010]_R//[\bar{1}10]_C$ and $(001)_R//(111)_C$, and **e,** along $[\bar{2}11]_C$ zone-axis demonstrating $[\bar{1}10]_R//[\bar{2}11]_C$ and $(001)_R//(111)_C$. Lattice parameters for both phases can be extracted from **d** with a = 6.15 Å for cubic GST, and a = 4.24 Å, c = 11.62 Å for rhombic SbTe. **f, g,** HAADF-STEM images with white arrows showing sub-unit-cell thick SbTe layers in the GST matrix, yellow arrows showing the Te-deficient atomic columns with weak intensity.

Rhombic SbTe nanophase in which Sb and Te atoms share a crystallographic site precipitated in the face-centered-cubic (FCC) GST matrix in the GST467 film. As shown in Fig. S8d-e, the SbTe nano-precipitate grows epitaxially on the $(111)_C$ plane along the $[111]_C$ direction. In contrast to the alternating arrangement of $(111)_{Te}$ and $(111)_{Ge/Sb}$ planes along the $[111]_C$ direction with equal crystal plane spacings in the GST FCC structure, SbTe has identical $(001)_{Sb/Te}$ planes with non-equal spacings along the $[001]_R$ direction, which is parallel to the $[111]_C$ direction. As the GST467 film has 50% higher Sb content than the Ge content, in the Sb-rich (Ge absence) local areas, the alternating arrangement of $(111)_{Te}$ and $(111)_{Sb}$ planes along the $[111]_C$ direction of cubic GST matrix favors the coherent precipitation of rhombic SbTe nanophase. The SbTe precipitate could be as thin as sub-unit-cell thick (Fig. S8f-g). As shown in Fig. S8g, the precipitation of SbTe nanophase from GST467 resulted in local Te-deficient (anion vacancy) structure nearby the SbTe precipitate, which could be stabilized by the interface strain of SbTe/GST. The stability of the GST467 nanocomposite is further reflected in extensively cycled GST467 PCM devices showing repeatable resistance vs. current and voltage profiles, as well as fast switching speed[8].

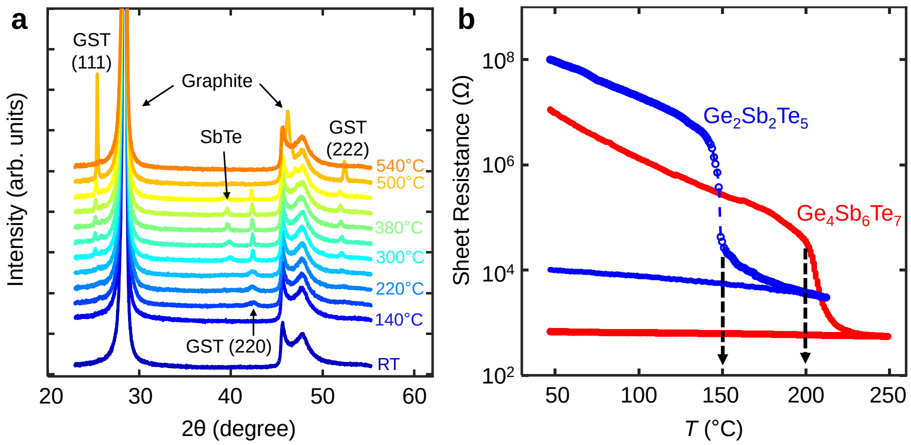

Supplementary Fig. S9 | a, X-ray diffraction (XRD) spectra of a $Ge_4Sb_6Te_7$ (GST467) film from room temperature (deposited in the amorphous state) to 540°C. Ge-Sb-Te diffraction peaks (labeled GST) emerge at ≈ 180 – 220 °C denoting the crystallization temperature. The disappearance of diffraction peaks at ≈ 540 °C indicates the melting temperature for GST467. Peaks from SbTe nanocomposites present in the GST467 film can also be seen. Some peaks are from the graphite dome covering the heating stage of the XRD measurement setup at high temperatures. **b**, Measured sheet resistance as a function of temperature for ≈ 200 nm thick GST467 (red) and GST225 (blue) films. Black dashed arrows indicate the crystallization temperatures, ≈ 150 °C for GST225 and ≈ 200 °C for GST467.

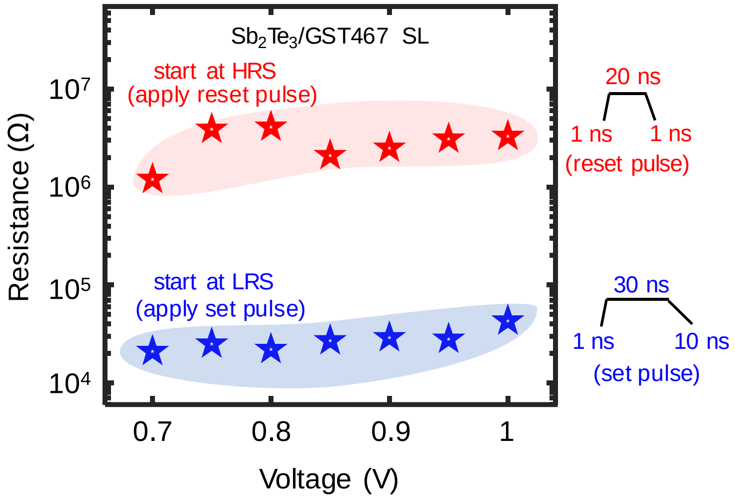

Supplementary Fig. S10 | Resistance vs. voltage in an Sb_2Te_3/GST467 superlattice PCM device with 40 nm BE diameter showing clear distinction between the low-resistance state (LRS) and the high-resistance state (HRS). While the difference in the reset and set pulse amplitude is not significant (≈ 0.1 - 0.2 V), their pulse fall times are very different (reset: 1/20/1 ns; set: 1/30/10 ns rise/width/fall time). Thus, the LRS and HRS states are separable from each other during the PCM operation. For example, a reset pulse (1/20/1 ns) with amplitude of 0.9 V (greater than the set voltage) does not induce an HRS-to-LRS transition because the falling edge is too short for crystallization to occur. On the other hand, a set pulse (1/30/10 ns) with amplitude of 0.9 V (greater than the reset voltage) does not induce an LRS-to-HRS transition because it does not have a sufficiently short falling edge (10 ns is the shortest falling edge we have used; longer falling edges also work, e.g., 50 ns). This distinguishes between LRS and HRS during device operation.

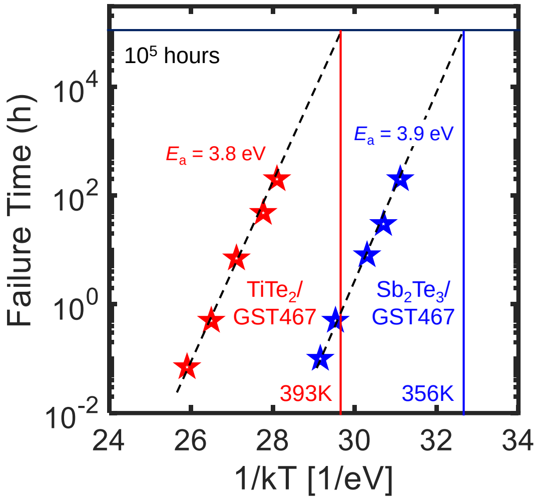

Supplementary Fig. S11 | Arrhenius plot for Sb_2Te_3/GST467 and $TiTe_2$/GST467 SL-PCM, showing failure time vs. 1/kT (k is the Boltzmann constant, T is the baking temperature). By extrapolation, the retention of Sb_2Te_3/GST467 and $TiTe_2$/GST467 SL-PCM is 10^5 hours at 356K and 393K, respectively. The activation energy of Sb_2Te_3/GST467 and $TiTe_2$/GST467 SL-PCM is 3.9 eV and 3.8 eV, respectively. Here we reset the cells and baked them at elevated temperatures until retention failure was observed (when cell resistance dropped below 100 kΩ).

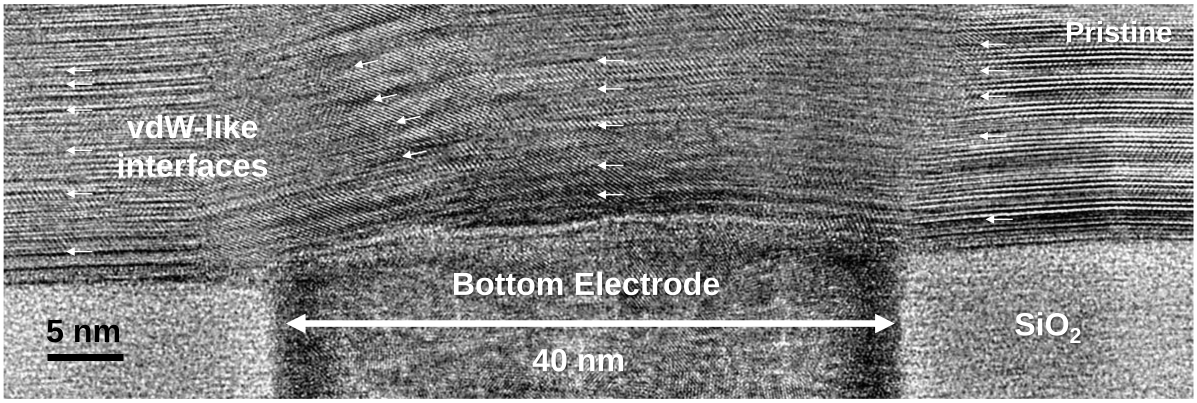

Supplementary Fig. S12 | High-resolution TEM cross-section (zoomed in near BE) of a $TiTe_2$/GST467 superlattice PCM pristine device (before any switching cycles) with 40 nm BE diameter.

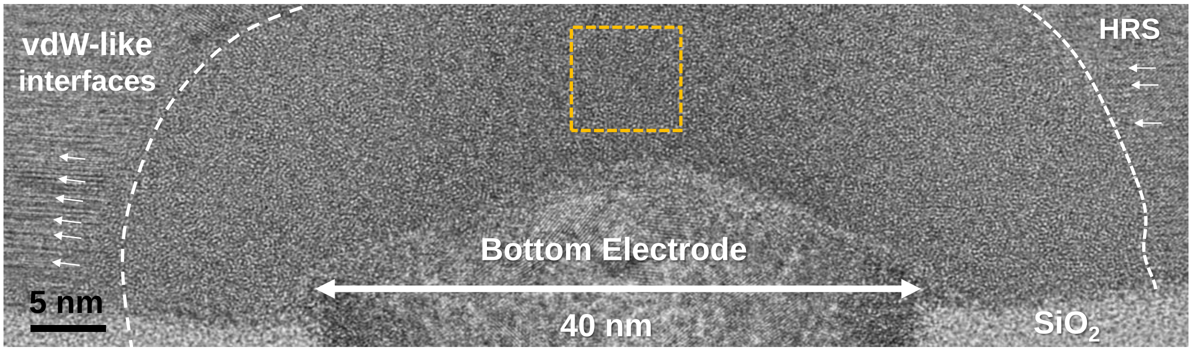

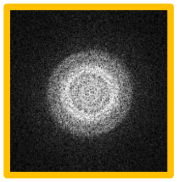

Supplementary Fig. S13 | High-resolution TEM cross-section (zoomed in near BE) of a $TiTe_2$/GST467 superlattice PCM with 40 nm BE diameter in the high-resistance state (after 10^4 times electrical cycling) showing the amorphous region surrounded by vdW-like interfaces, similar to Sb_2Te_3/GST467 SL-PCM (Fig. S1). We infer the possible presence of nano-crystallites[1,2] within this amorphous region of $TiTe_2$/GST467 superlattice, as evidenced by the diffraction rings (inset) which might further facilitate the fast-switching[2,3] of these devices. We note that reset pulses can lead to partial amorphization near the bottom electrode of our superlattice-like devices. In the subsequent set operation, these regions can act as a template to re-construct the vdW-like gaps within the active region[5,9].

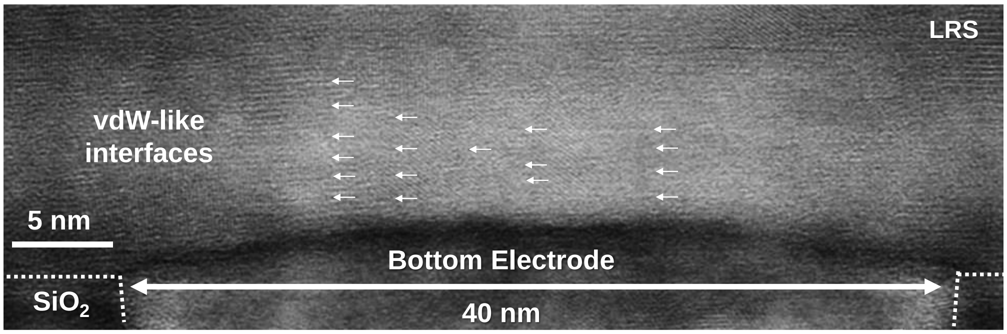

Supplementary Fig. S14 | High-resolution TEM cross-section (zoomed in near BE) of a cycled $TiTe_2$/GST467 superlattice PCM with 40 nm BE diameter in the low resistance state. The TEM shows the presence of vdW-like gaps in $TiTe_2$/GST467 superlattice PCM, similar to Sb_2Te_3/GST467 SL (Supplementary Fig. S2).

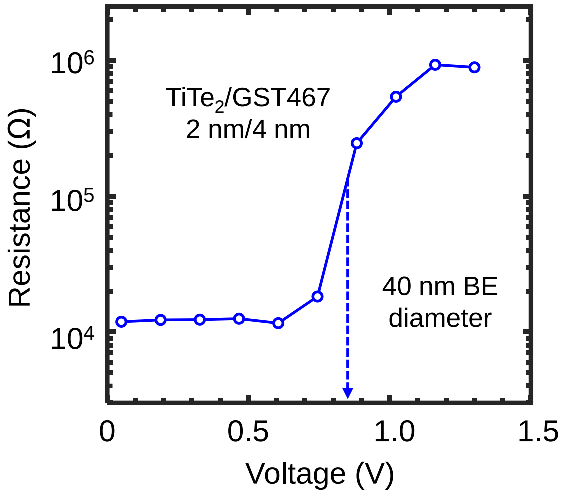

Supplementary Fig. S15 | Read resistance vs. voltage for a $TiTe_2$/GST467 superlattice PCM device with 40 nm BE diameter showing sub-1 volt (≈ 0.85 V) reset voltage. Reset voltage (marked by blue dashed arrow) is defined as the voltage needed for a ≈ 10× resistance increase from LRS.

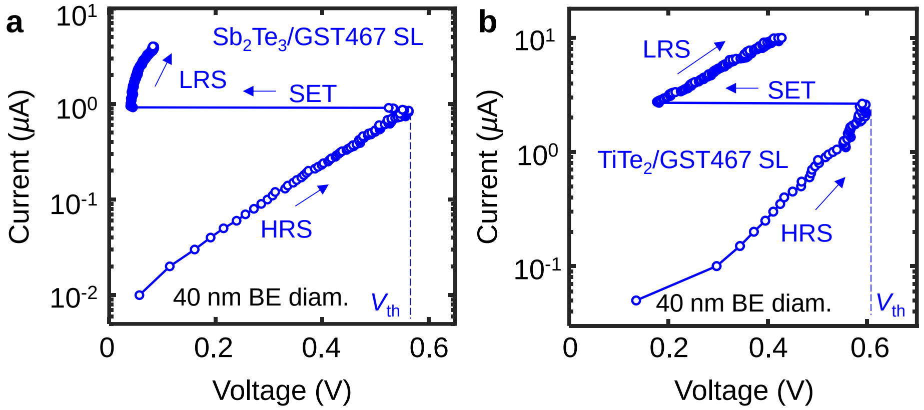

Supplementary Fig. S16 | Current vs. voltage for **a**, Sb_2Te_3/GST467 and **b**, $TiTe_2$/GST467 superlattice PCM devices (both with 40 nm BE diameter) showing threshold switching behavior, similar to the thermally driven melt-quench based phase transition in conventional PCM[10].

References:

1. Ding, K. *et al.* Low-Energy Amorphization of $Ti_1Sb_2Te_5$ Phase Change Alloy Induced by $TiTe_2$ Nano-Lamellae. *Sci. Rep.* **6**, 30645 (2016).
2. Xia, M. *et al.* Ti-Sb-Te Alloy: A Candidate for Fast and Long-life Phase-change Memory. *ACS Appl. Mater. Interfaces* **7**, 7627 (2015).
3. Liu, Y.-G. *et al.* Fast Switching and Low Drift of TiSbTe Thin Films for Phase Change Memory Applications. *Mater. Sci. Semicond. Process.* **91**, 399 (2019).
4. Kwon, H. *et al.* Uncovering Thermal and Electrical Properties of Sb_2Te_3/GeTe Superlattice Films. *Nano Lett.* **21**, 5984–5990 (2021).
5. Khan, A. I. *et al.* Electro-Thermal Confinement Enables Improved Superlattice Phase Change Memory. *IEEE Electron Device Lett.* **43**, 204–207 (2022).
6. Khan, A. I. *et al.* Unveiling the Effect of Superlattice Interfaces and Intermixing on Phase Change Memory Performance. *Nano Lett.* **22**, 6285–6291 (2022).
7. Khan, A. I. *et al.* Ultralow–switching Current Density Multilevel Phase-change Memory on a Flexible Substrate. *Science (80-.).* **373**, 1243–1247 (2021).
8. Khan, A. I. *et al.* Energy Efficient Neuro-inspired Phase Change Memory Based on $Ge_4Sb_6Te_7$ as a Novel Epitaxial Nanocomposite. *Adv. Mater.* **7**, 2300107 (2023).
9. Boniardi, M. *et al.* Evidence for Thermal-Based Transition in Super-Lattice Phase Change Memory. *Phys. status solidi – Rapid Res. Lett.* **13**, 1800634 (2019).
10. Le Gallo, M., Athmanathan, A., Krebs, D. & Sebastian, A. Evidence for Thermally Assisted Threshold Switching Behavior in Nanoscale Phase-change Memory Cells. *J. Appl. Phys.* **119**, 25704 (2016).
